# Trans-Pacific Partnership Provisions in Intellectual Property, Transparency, and Investment Chapters Threaten Access to Medicines in the US and Elsewhere

**DOI:** 10.1371/journal.pmed.1001970

**Published:** 2016-03-08

**Authors:** Brook K. Baker

**Affiliations:** 1 Northeastern University School of Law, Program on Human Rights and the Global Economy, Boston, Massachusetts, United States of America; 2 University of KwaZulu Natal, Durban, South Africa; 3 Health Global Access Project, New York, New York, United States of America

## Abstract

Brook Baker describes the potential harms to global health from the Trans Pacific Partnership Agreement and its failure to balance the interests of patients and the public with those of industry.

**Summary Points**

- The recently negotiated Trans Pacific Partnership Agreement (TPP) contains provisions that would dramatically and negatively impact access to affordable medicines in the United States and elsewhere if it is ratified.
- Provisions in the Intellectual Property (IP) Chapter of TPP lengthen, broaden, and strengthen patent-related monopolies on medicine and erect new monopoly protections on regulatory data as well. IP Chapter enforcement provisions also mandate injunctions preventing medicines sales, increase damage awards, and expand confiscation of medicines at the border.
- IP rightholders gain new powers in the Investment Chapter to bring private, IP-related investor-state-dispute-settlement (ISDS) damage claims directly against foreign governments before unreviewable, three-person arbitration panels. Unrestricted IP-investor damage claims deter countries’ willingness to render adverse IP decisions and to adopt IP policy flexibilities designed to increase access to affordable medicines.
- The Transparency Chapter contains provisions that allow pharmaceutical companies more access to government decisions listing medicines and medical devices for reimbursement.
- At the very least, these multiple TPP provisions that extend pharmaceutical powers should be scaled back to the minimum consensus standards reached in the 1994 World Trade Organization (WTO) Trade Related Aspects of Intellectual Property Rights (TRIPS) Agreement. Health advocates should convince the US Congress and opponents in other countries to reject an agreement that could so adversely impact access to medicines.

## Introduction

A new Pacific-Rim trade agreement threatens future access to affordable medicines in the United States and abroad. Buried in 6,000-plus pages of text, annexes, and side letters, there are multiple provisions—complex in their articulation, but simple in their effect: they dramatically increase monopoly protections for the transnational originator pharmaceutical industry.

With the Trade Promotion Authority, a procedure for accelerating congressional approval of trade agreements, enacted after a brutal and circuitous congressional battle in June [1], parties to the Trans-Pacific Partnership Agreement (TPP) finally reached a draft agreement on October 4, 2015 [2], and released the text one month later [3]. This trade pact, negotiated by the US for more than five years, involves 12 countries controlling nearly 40% of the global economy. The biggest players are the US and Japan, but there is a mix of other rich and middle-income countries, including Australia, Brunei, Canada, Chile, Malaysia, Mexico, New Zealand, Peru, Singapore, and Vietnam. Critical issues affecting future access to affordable medicines are at the center of the agreement.

One of the most contentious chapters in the TPP is the Intellectual Property (IP) Chapter [4], whose patent, undisclosed-test-or-other-data, and enforcement provisions could dramatically affect access to affordable medicines. Equally contentious is the Investment Chapter [5], in which protection of IP-related investments and investor-state-dispute settlement (ISDS) give drug companies powerful tools to protect monopolies on medicines. An annex to the Transparency Chapter [6] risks negatively impacting access to medicines because of increased applicant access to medical product listing decisions. TPP negotiations had been controversially conducted in strict secrecy (except for hundreds of cleared industry advisors on trade advisory committees), with zero official public access to proposed texts [7]; even members of Congress complained about their limited access [8].

TPP parties have been negotiating against a backdrop where there has already been global harmonization of minimum standards of protections for pharmaceutical IP rightholders. In 1994, countries adopted the World Trade Organization (WTO) Agreement on Trade Related Aspects of Intellectual Property Rights (TRIPS) [9], which established baseline, harmonized standards for the protection and enforcement of patents, trademarks, copyrights, and trade secret regulatory/clinical trial data. With respect to patent rights, TRIPS provides for a minimum term of 20 years of patent protection and prohibits countries from excluding patents on pharmaceutical products, a policy position previously held by nearly fifty countries, including India and Brazil. Although it is true that TRIPS prescribes minimum IP protections, it also outlines key public health flexibilities—in other words, it partially balances the rights of inventors and creators, users, and the public-at-large. In particular, least-developed and developing countries were given transition periods within which to become TRIPS-compliant, and all WTO members have rights to issue compulsory licenses (allowing some degree of generic competition), to allow parallel importation (buying abroad at a cheaper price), to define more stringent standards of patentability (reducing frivolous and secondary patents), and to adopt exemptions, limitations, and exceptions to IP rights (such as research rights and early-working/Bolar rights).

As revealed in earlier leaked drafts of the TPP, the most protective, TRIPS-plus proposals for pharmaceutical rightholders are those put forward by the office of the US Trade Representative (USTR). The USTR touted its IP proposals as being good for US exports, for jobs in its creative industries, and for innovation of new technologies. Although some troubling proposals were removed from the final text, like provisions mandating patents on new forms of existing medicines and prohibitions against patent opposition procedures, the TPP still contains many provisions that restrict TRIPS-compliant policy space to improve the quality of patents and to use flexibilities to bypass patent and other IP monopolies to encourage generic competition and other cost-controlling and research-enhancing measures.

## Analysis

### The TPP’s IP Chapter Imposes Longer, Broader, and Stronger Patent Monopolies on Medicines

The TPP’s IP Chapter contains final provisions that (1) weaken standards of patentability, leading to more patents; (2) extend patent terms to compensate for delays in granting patents or registering marketing approval; (3) adopt new exclusive rights relating to undisclosed registration-related/clinical-trial data; (4) prevent or interfere with the registration of generics where patents are claimed; and (5) enhance patent infringement remedies.

In general, weak standards of patentability, the criteria upon which patents are granted, make it easier to get initial patents—the first 20 years of exclusive rights; weak standards also encourage seeking multiple secondary patents that further extend the period of exclusivity. Using weak patent standards, drug companies can obtain “secondary,” 20-year patents on minor variations of an active ingredient, new formulations and dosages, new uses or methods of use (indications), and new processes of synthesis and manufacture. In the pharmaceutical industry, pursuit of secondary patents is positively framed as patent life-cycle management; in the access-to-medicines world it is criticized as manipulative evergreening [10].

The TPP imposes weakened standard of patentability in two ways. First, it defines “obviousness,” the real meat of the inventiveness inquiry, to eliminate countries’ right to require that an invention have a significant technological advantage as assessed by persons “highly” skilled in the relevant arts. Second, the TPP mandates that countries allow patents on new uses or methods or process of use of known medicines, even though research and development costs are significantly lower for a medicine already known to be safe and even though the search for new indications is more a function of routine, plodding investigation than “inventive-step” science [11]. The consequences of weak standards of patentability can be quite significant. For example, there are over 800 different families of patents on the antiretroviral booster, ritonavir [12], and its period of exclusivity had been extended for decades [13]. Such extended periods of exclusivity can have significant cost implications [14].

The TPP lengthens pharmaceutical monopolies in other ways. For example, if there is an unreasonable delay in the granting of a patent (within five years of the filing of a patent application or within three years after a request for patent examination) then the patent term must be adjusted to compensate for the delay. In addition, the TPP requires patent terms adjustments to compensate for delays in the marketing approval process. A study from the US on the impact of patent term extensions found that they add an average of 3.6 years to the period of exclusivity and might account for nearly 20% of pharmaceutical sales in the US [15].

The TPP creates additional forms of monopoly protection by requiring countries to adopt data/marketing exclusivity restrictions like those applied in the US [16]. Accordingly, when a pharmaceutical product involving a new chemical entity receives marketing approval, the relevant drug regulatory authority and generic applicants cannot refer to or rely on the undisclosed regulatory data submitted by the originator, nor on the fact of the prior registration, to assess the therapeutic equivalence of the follow-on product for a minimum period of five years. This period of exclusivity can be extended by successive three-year periods each time the originator submits additional clinical information for a new use of the medicine. The TPP also imposes US-style patent-registration linkage (blockage of registration by the medicines regulatory authority), or requires notice to patent holders and timely access to judicial or administrative procedures for preventing market approval of a follow-on generic equivalent whenever a product patent is claimed.

An even longer period of data/marketing exclusivity is required for biologics. Although there is no economic evidence justifying longer exclusivity for biologics [17], the US fought hard for twelve years of exclusivity. The parties settled on eight years of data exclusivity for biologics or five years followed by three years of equivalent market protection. Because biologics are a growing proportion of the market and are often significantly more expensive than small-molecule medicines, expanding exclusivity can have significant cost implications. There are several studies showing that data exclusivity raises prices and negatively impacts access to medicines [18].

The Enforcement Section of the IP Chapter strengthens private enforcement of IP rights and imposes greater enforcement obligations on governments. It contains provisions requiring deterrent remedies, compelling the use of the rightholder’s retail price as a measure of damages, mandating injunctive relief, and banning reasonable royalties as an infringement remedy. Several of these proposals exceed US law [19]. TPP governments will also be required to adopt border control measures like those that interrupted lawful passage of generic medicines through Europe in 2008 and 2009 [20]. Fear of excess liability, injunctions, and border seizures can deter generics from marketing competing equivalents when there is even a slight risk of patent infringement enforcement.

### The Investment Chapter Grants Additional Enforcement Powers to IP Rightholders

In the Investment Chapter, IP rights are defined as protected investments, and foreign IP investors are permitted to seek private arbitration through ISDS whenever they feel that their investments have been treated unfairly or inequitably, taken without compensation, or discriminated against. TPP member states can expect an avalanche of IP-related claims from disappointed pharmaceutical companies that think their legitimate expectations of future profits have been thwarted by foreign governments’ IP decisions or policies.

The TPP’s Investment Chapter greatly expands the enforcement rights of foreign pharmaceutical companies, creating substantial risks to countries’ ability to set IP-related policy and to render IP decisions. The Investment Chapter unequivocally defines IP rights as “investments.” It prohibits the following: (1) discrimination against foreign IP investors, (2) unfair and inequitable treatment, and (3) indirect expropriation. More pointedly, it allows ISDS claims directly against governments before unreviewable three-person arbitration panels, even when judicial remedies have not been exhausted or when companies have lost on appeal. Foreign investors can bring ISDS claims that domestic investors cannot. Moreover, companies might claim—correctly or not—a lack of fair and equitable treatment that undermines their well-grounded expectations of profit with respect to many health-related regulatory and judicial decisions, including the following: denials or revocations of pharmaceutical patents; granting of compulsory licenses; denials or restrictions on marketing rights; refusals to list excessively priced, IP-protected products for reimbursement; decisions to establish price controls; and required disclosure of clinical trial data. Foreign companies might claim indirect expropriation following changes in regulatory environments, including changes designed to promote public health [21].

The dangers of ISDS IP enforcement are highlighted in a US$500 million arbitration claim brought by Eli Lilly against the government of Canada under the North America Free Trade Agreement because it revoked patents on two medicines that Canada’s highest court had found failed to satisfy well-established standards of patentability in Canada [22]. Canada will have to spend millions of dollars to defend against this claim even if it ultimately wins, which is likely. The even greater danger is that other, poorer countries will be intimidated away from regulating or otherwise acting against the interests of foreign IP investors, even if they are doing so in a non-discriminatory fashion and in the interests of public health.

### The Transparency Chapter on Pharmaceuticals and Medical Devices Increases Industry’s Role in Medical Reimbursement Listings

In the Transparency Chapter Annex addressing transparency and procedural fairness for pharmaceutical products and medical devices, companies are given multiple opportunities to intercede in decisions to list products for reimbursement. These interventions could result in more listings of higher-priced medicines even in the absence of convincing evidence of added therapeutic value.

Under the Pharmaceutical Product and Medical Device Transparency Annex, companies will have multiple chances to influence pharmaceutical/medical-device listing decisions, to scrutinize resulting decisions, and to challenge decisions previously rendered. These multiple inputs can result in more listings, higher prices, and higher administrative costs for affected countries. The Transparency Chapter also gives other countries direct opportunities to complain about individual listing decisions, patterns and practices of decisions, and decision-making criteria and processes.

## Discussion

Medical professionals, patients, and others concerned with access to medicines and enhanced generic competition might wonder whether there are justifications for a trade agreement that strengthens IP, investment, and regulatory-participation rights for the originator pharmaceutical industry. They might also wonder what alternative provisions should be in the TPP. The historic justification for longer, stronger, and broader IP protections for medicines is quite simple—they give rise to economic power to charge what the market will bear so that companies can earn extra profits that they thereafter invest to invent the next generation of life-enhancing medicines, including those for currently untreated or undertreated conditions [23]. As successful as the IP system might be for doing so—and this is highly disputed [24]—it surely comes at an enormous cost, one that is increasingly intolerable not only to US patients and payers but even more so to poorer populations and poor governments. This analysis concludes, at the very least, that the TPP should not require greater patent, data, and enforcement protections than those prescribed by TRIPS, and instead that it should clarify and endorse greater adoption and use of allowable flexibilities. An even more far-reaching approach, one that cannot be fully developed here, would encourage greater government investment in and regulation of research and development (R&D) with the aim of fully supporting innovation, including clinical trials, while at the same time preserving policy space to ensure competitive access to new medical technologies and their rapid dissemination throughout the world. If one regards medicines as global public goods [25] and agrees that access to the tools of health create both individual and communal benefits, then one might support trade policies that delink the need for R&D resources from monopoly protections that result in access-prohibitive prices [26], not only for developing countries but increasingly for rich countries as well.

IP maximization in the TPP will harm access to more affordable medicines in both the US and its trading partners. Policy space on both sides of the Pacific will be reduced while opportunities for excessive pricing will increase dramatically with predictable adverse consequence for the right to health. Armed with knowledge about the details of the TPP’s anti-access provisions, there is still time for health advocates to convince the US Congress and TPP partners that the TPP’s monopoly-enhancing measures must be rejected.

## Abbreviations

IP: Intellectual Property
ISDS: investor-state-dispute-settlement
R&D: research and development
TPP: Trans Pacific Partnership Agreement
TRIPS: Trade Related Aspects of intellectual Property Rights
USTR: US Trade Representative
WTO: World Trade Organization

## References

[1] Boyer D. Obama signs trade legislation; Bonner, McConnell miss White House Event. The Washington Times. 2015 June 29. http://www.washingtontimes.com/news/2015/jun/29/boehner-mcconnell-skip-event-obama-sign-trade-bill/. Accessed 17 December 2015.

[2] Office of the United States Trade Representative, Press Release: Summary of the Trans Pacific Partnership Agreement (Oct. 4, 2015). https://ustr.gov/about-us/policy-offices/press-office/press-releases/2015/october/summary-trans-pacific-partnership.

[3] Trans Pacific Partnership Agreement. https://ustr.gov/trade-agreements/free-trade-agreements/trans-pacific-partnership/tpp-full-text.

[4] Chapter 18: Intellectual Property. https://ustr.gov/sites/default/files/TPP-Final-Text-Intellectual-Property.pdf.

[5] Chapter 9: Investment. https://ustr.gov/sites/default/files/TPP-Final-Text-Investment.pdf.

[6] Annex 26-A: Transparency and Procedural Fairness for Pharmaceutical Products and Medical Devices. https://ustr.gov/sites/default/files/TPP-Final-Text-Transparency-and-Anti-corruption.pdf.

[7] Kaminski M. Op-Ed: Don’t Keep the Trans-Pacific Partnership Talks Secret, The New York Times. 2015 April 24. http://www.nytimes.com/2015/04/14/opinion/dont-keep-trade-talks-secret.html. Accessed 17 December 2015.

[8] Dovere E-I. Extreme secrecy eroding support for Obama’s trade pact. Politico. 2015 May 4. http://www.politico.com/story/2015/05/secrecy-eroding-support-for-trade-pact-critics-say-117581. Accessed 17 December 2015.

[9] World Trade Organization. Agreement on Trade-Related Aspects of Intellectual Property Rights, Marrakesh Agreement Establishing the World Trade Organization, Annex 1C, Art. 8(1), 33 I.L.M. 81. 1994.

[10] Bansal IS, Sahu D, Bakshi G, Singh S. Evergreening: A Controversial Issue in Pharma Milieu. J Intel Prop Rgts. 2009 7; 14: 299–306.

[11] Correa C. Guidelines for Examination of Pharmaceutical Patents from a Public Health Perspective. ICTSD, WHO, UNCTAD; 2007 http://www.ufrgs.br/antropi/lib/exe/fetch.php?media=correa_pharmaceutical-patents-guidelines.pdf.

[12] WIPO, Patent Landscape for Ritonavir. 2011. http://www.wipo.int/edocs/pubdocs/en/patents/946/wipo_pub_946.pdf.

[13] Amin T, Kesselheim AS. Secondary Patenting of Branded Pharmaceuticals: A Case Study of How Patents on Two HIV Drugs Could be Extended for Decades. Heath Affairs. 2012; 31: 2286–94. 10.1377/hlthaff.2012.0107 23048110

[14] Vernaz N, Haller G, Girardin F, Huttner B, Combescure C, Dayer P, et al Patent Drug Extension Strategies on Healthcare Spending: A Cost-Evaluation Analysis. PLoS Med. 2013; 10: e1001460 doi: 10.1371/journal.pmed.1001460 23750120 10.1371/journal.pmed.1001460 PMC3672218

[15] Clift C. The Value of Patent Term Extensions to the Pharmaceutical Industry in the USA. J Gen Medicines. 2008; 5: 201–08.

[16] Baker BK. Ending drug registration apartheid–taming data exclusivity and patent/registration linkage. Am J Law & Med. 2008; 34: 303–44. 18697696 10.1177/009885880803400209

[17] Federal Trade Commission. Emerging Health Care Issues: Follow-on Biologic Drug Competition. June 2009. https://www.ftc.gov/sites/default/files/documents/reports/emerging-health-care-issues-follow-biologic-drug-competition-federal-trade-commission-report/p083901biologicsreport.pdf.

[18] Chakrabarti G. Need of Data Exclusivity: Impact on Access to Medicines. J Intel Prop R. 2014; 19: 325–36.

[19] Love J. Inside Views: The TPP’s Reckless Proposals For Damages Will Have Negative Impact On Future Reform Of IPR Regimes [blog]. IP-Watch. July 28, 2015. http://www.ip-watch.org/2015/07/28/the-tpps-reckless-proposals-for-damages-will-have-negative-impact-on-future-reform-of-ipr-regimes/.

[20] Baker BK. Settlement of India/EU WTO Dispute re Seizures of In-Transit Medicines: Why the Proposed EU Border Regulation Isn't Good Enough. PIJIP Res Papers Series no. 2012–02 American University Washington College of Law, Washington, D.C. http://digitalcommons.wcl.american.edu/cgi/viewcontent.cgi?article=1026&context=research.

[21] Baker B, Geddes K. Corporate Power Unbound: Investor-State Arbitration of IP Monopolies on Medicines–*Eli Lilly v* . *Canada* and the Trans-Pacific Partnership Agreement. J Intel Prop L. 2016; 23: in press.

[22] Eli Lilly and Company v. The Government of Canada, UNCITRAL, ICSID Case No. UNCT/14/2. http://www.italaw.com/cases/1625.

[23] Pharmaceutical Research and Manufacturers of America. 2015 Profile: Pharmaceutical Research Industry. 2015. http://www.phrma.org/sites/default/files/pdf/2015_phrma_profile.pdf.

[24] Light D., Warburton R. Demythologizing the high costs of pharmaceutical research. Biosocieties. 2011; 6:34–50. http://www.pharmamyths.net/files/Biosocieties_2011_Myths_of_High_Drug_Research_Costs.pdf.

[25] Moon S. Medicines as Global Public Goods: The Governance of Technological Innovation in the New Era of Global health. Glob H Gov. 2008–09; II: 1–23.

[26] Love J. Balancing Elements for Health Research and Development. Bull WHO. 2012; 90: 796–796A. doi: 10.2471/BLT.12.113886 23226887 10.2471/BLT.12.113886 PMC3506415

